# Cellular Delivery of Quantum Dot-Bound Hybridization Probe for Detection of Intracellular Pre-MicroRNA Using Chitosan/Poly(γ-Glutamic Acid) Complex as a Carrier

**DOI:** 10.1371/journal.pone.0065540

**Published:** 2013-06-07

**Authors:** Yao Geng, Dajie Lin, Lijia Shao, Feng Yan, Huangxian Ju

**Affiliations:** 1 Jiangsu Institute of Cancer Prevention and Cure, Nanjing, China; 2 State Key Laboratory of Analytical Chemistry for Life Science, School of Chemistry and Chemical Engineering, Nanjing University, Nanjing, China; National Cancer Institute at Frederick, United States of America

## Abstract

A quantum dot (QD)-bound hybridization probe was designed for detection of intracellular pre-miRNA using chitosan (CS)/poly(γ-glutamic acid) (γ-PGA) complex as a gene vector. The probe was prepared by assembling thiolated RNA to gold nanoparticle (Au NP) via Au-S bond and then binding 3′-end amine of the RNA to the carboxy group capped on quantum dot surface. The QD-RNA-Au NP probe was assembled on the vector by mixing with aqueous γ-PGA solution and then CS solution to construct a gene delivery system for highly effective cellular uptake and delivery. After the probe was released from CS/γ-PGA complex to the cytoplasm by electrostatic repulsion at intracellular pH, it hybridized with pre-miRNA precursor as target. The formed product was then cleaved by RNase III Dicer, leading to the separation of QDs from Au NPs and fluorescence emission of QDs, which could be detected by confocal microscopic imaging to monitor the amount of the intracellular pre-miRNA precursor. The in vitro assays revealed that the QD-RNA-Au NP was a robust, sensitive and selective probe for quantitative detection of target pre-miRNA. Using MDA-MB231 and MCF-7 breast cancer cells as models, the relative amount of pre-miRNA let-7a could be successfully compared. Since the amount of miRNA is related to the progress and prognosis of cancer, this strategy could be expected to hold promising application potential in medical research and clinical diagnostics.

## Introduction

MicroRNAs (miRNAs) are a family of small non-coding RNA molecules of plants and animals. Mature miRNAs generally contain 19 to 25 nucleotides that are processed from hairpin pre-miRNA precursors containing 60 to 100 nucleotides [1], [2]. In the cytoplasm, the precursor hairpins can be recognized and then cleaved by the RNase III Dicer into small imperfect dsRNA duplexes to produce mature miRNAs after one strand is degraded [3], [4], [5]. These miRNAs play important roles in various cellular and disease processes [6] and have been suggested as a novel class of oncogenes or tumor suppressor genes [7]. Unique patterns of altered miRNA expression in cancer cells can serve as a diagnostic fingerprint [8].

Among the discovered tumor-related miRNAs, the down-regulation of miRNA let-7a expression has been found in breast cancer samples with either lymph node metastasis or higher proliferation index, suggesting that a reduced let-7a expression can be associated with a poor prognosis [9]. An association between let-7a down-regulation and poor prognosis has also been reported in human lung cancer [10]. Thus, miRNA let-7a may be a good candidate as biomarker to promise diagnostic, prognostic, and predictive information of cancer [11]. Both in vitro and in vivo analyses of miRNA let-7a have attracted significant attention. Due to the exceptionally short length, very similar nucleotide sequences and quite low expression levels, miRNA analysis requires improved miRNA profiling techniques. The current techniques predominantly include PCR [12], microarray analysis [13] and Northern blotting [14]. These technologies are expensive and time-consuming, and cannot be used for in situ or intracellular miRNA analysis. Recently, several miRNA detection methods based on alternative biosensing techniques, such as nanoparticle-amplified surface plasmon resonance imaging or bioluminescence detection, have been reported [15], [16], [17]. These methods still have a limitation that they require miRNA enrichment followed by labeling of miRNA isolated from the sample prior to detection. Our previous works proposed two cellular delivery systems using polyethylenimine-grafted graphene nanoribbon [18] and multifunctional SnO2 nanoparticles (NPs) [19] as gene vectors to present two methods for target-cell-specific imaging and detection of intracellular pre-miRNA by fluorescence resonance energy transfer (FRET). These works indicated that both the probe itself and the cellular delivery system with good biocompatibility, low immunogenicity, reduced cytotoxicity, particularly, the high efficiency of cellular uptake and delivery are the necessary protocol to achieve in situ or intracellular miRNA analysis.

**Figure 1:**
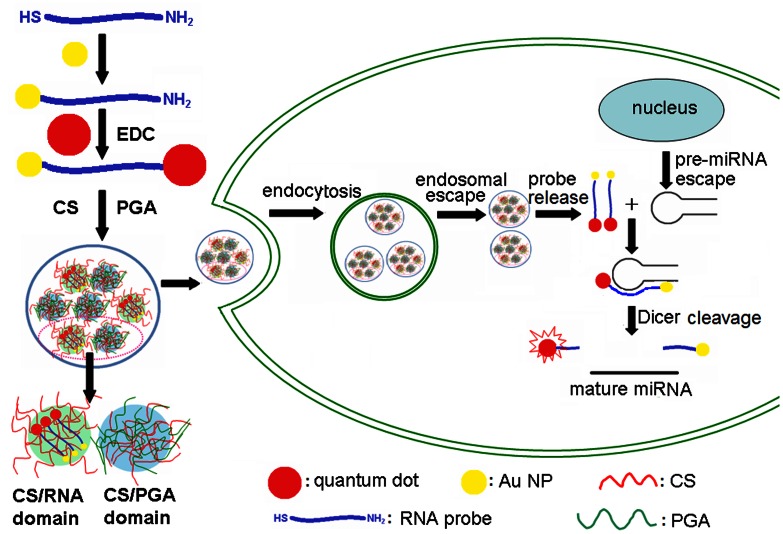
Transfection of QD-RNA-Au NP probe into cell via CS/γ-PGA complex for imaging of intracellular pre-miRNA.

Chitosan, as a biocompatible, low-immunogenic and cytotoxic polymer [20], [21], [22], has been considered as an excellent candidate for various biomedical applications such as drug delivery, tissue engineering and gene delivery [23], [24], [25], [26]. Some chitosan-based complexes have been presented to improve the transfection efficiency for gene delivery. By incorporating a negatively charged poly(γ-glutamic acid) (γ-PGA) in CS/DNA complex, the cellular uptake can be signficantly enhanced [27]. Thus CS/γ-PGA complex has been used to transfect miRNA into cells and assist the intracellular release of miRNA for enhancing the effectiveness in gene silencing [28]. The following study has shown that a free γ-glutamic acid in the N-terminal end of CS/γ-PGA complex can be easily recognized by γ-glutamyl transpeptidase in the cell membrane to significantly increase the cellular uptake [29]. This work combined the advantages of CS/γ-PGA complex with the fluorescence emission of quantum dots (QDs) [30], [31] to construct a gene delivery system for highly effective imaging monitoring of intracellular miRNA using a newly designed QD-bound hybridization probe. Here QD were bound to the 3′-end amine of RNA probe that was assembled on Au NP via Au-S bond. The FRET from QD to Au NP quenched the photoluminescence of QDs. After delivered into living cells, the QD-bound RNA probe was released from the complex at intracellular pH to hybridize with pre-miRNA precursor, and the formed product was cleaved by RNase III Dicer to separate the QD and Au NP, leading to the detectable fluorescence emission of QDs (Figure 1). The proposed probe and method provided a new avenue for monitoring the intracellular pre-miRNA precursors, showing promising application in biomedicine.

## Materials and Methods

### Materials and Reagents

Low molecular weight chitosan (CS) was purchased from TCI Co. Ltd (Shanghai, China). 1-Ethyl-3-(3-dimethylaminopropyl) carbodiimide hydrochloride (EDC) was obtained from Sigma (St. Louis, MO, USA). Poly-γ- glutamic acid (γ-PGA, 15–70 kD) was from Saitaisi Co. Ltd (Nanjing, China). Carboxyl ZnS/CdSe quantum dots (Qdot® 705 ITK™) was purchased from Invitrogen (Shanghai, China), which have narrow, symmetric emission bands with emission maxima near 705 nm. ShortCut® RNase III was purchased from New England Biolabs Ltd (Beijing, China). 3-(4,5-Dimethylthiazol-2-yl)-2,5-diphenyl-tetrazolium bromide (MTT) and sodium dodecyl sulfate (SDS) were from KeyGen Biotech. Co. Ltd. (Nanjing, China). The oligonucleotides were purchased from Sangon Biological Engineering Technology & Co. Ltd (Shanghai, China) and purified using high-performance liquid chromatography. Their sequences were:

probe RNA: 5′SH- AAUAUUCCAAACUAAUACAACCUCAUACCUCA-NH_2_3’.

control probe: 5′SH- AAUAUUUCACCCUAUGACAACCGACGACCUCA-NH_2_3’.

target pre-miRNA: 5′UGAGGUAGUAGGUUGUAUAGUUUGGAAUAUUACCACG3’.

### Synthesis of QD-RNA-Au NP Probe

The QD-RNA-Au NP probe was synthesized according to previous report with some modification [30], [33], [34]. Firstly, citrate stabilized Au NPs were prepared using the Frens method [35] and rendered RNase-free by treatment with 0.1% diethylpyrocarbonate (DEPC, Sigma). After adding probe RNA (20 µM) and stirring at room temperature for 2 h, The obtained mixture was filtrated through a PTFE membrane (0.45-µm pore size), and the solid was stirred in 5 ml of water for 30 min. Afterwards, QDs and EDC were added into the mixture and stirring at room temperature for 1 h, the mixture was then filtered through a PTFE membrane (0.45-µm pore size), the QD-RNA-Au NP probe was dispersed in 5 ml of DEPC-treated water and stirred for 30 min, which was stored at 4°C until use. As comparison, QD-control RNA-Au NP probe was synthesized with the same procedure.

### Preparation of Probe Loaded CS/γ-PGA Complex

The probe loaded CS/γ-PGA complex was prepared according to a simple complexation procedure as described previously [29], [36], [37]. Briefly, chitosan was dissolved in sodium acetate buffer (0.2 M, pH 6.0) and passed through a 0.22 µm filter to obtain a 50 µg/mL chitosan stock solution. 10 µg/mL γ-PGA solution was prepared in DEPC-treated water. 20 µl QD-RNA-Au NP (20 µM) was mixed with 20 µl γ-PGA and diluted to 100 µl with DEPC-treated water. After 100 µL chitosan solution was added, the mixture was immediately vortexed for 1 min, and left at room temperature for 1 h to obtain the solution of probe loaded CS/γ-PGA complex. With the same way, the control system was prepared. The loading of QD-RNA-Au NP probe on CS/γ-PGA was evaluated by a gel retardation assay, which was carried out using a 2% agarose gel with a current of 100 V for 30 min in TAE buffer solution (40 mM Tris HCl, 1% acetic acid and 1 mM EDTA).

### Cell Culture

MDA-MB-231 and MCF-7 breast cancer cell lines were kindly provided by the Jiangsu Institute of Cancer Prevention and Cure, Nanjing, China. Cells were cultivated in Dulbecco's modified Eagle's medium (DMEM, GIBCO) supplemented with 10% fetal calf serum, penicillin (100 µg/ml) and streptomycin (100 µg/ml) at 37°C in a humidified 5% CO_2_-containing atmosphere. The cell numbers were obtained using a Petroff-Hausser cell counter (USA). Human vascular endothelial cells (EC-304) were used as control.

### In vitro Detection of Pre-miRNA

10 µl of the probe loaded CS/γ-PGA complex was mixed with 490 µl target pre-miRNA for hybridization at room temperature for 15 min. The mixture was then treated with RNase III (1 unit) for 30 min to record the fluorescence spectrum on RF-5301 PC (SHIMDZU).To verify the results of intracellular pre-miRNA analysis, the expression levels of the pre-miRNA precursors in MDA-MB-231 and MCF-7 breast cancer cells were determined using real-time quantitative PCR, which was performed using an ABI Prism 7900 Sequence Detection System (Perkin-Elmer Applied Biosystems, Foster City, CA), the SYBR Green PCR Master Mix (Perkin-Elmer Applied Biosystems), and random-primed cDNAs (corresponding to 20 ng of total RNA extracted from cell lines). β-actin was used as control gene, and the primer pairs of miRNA let-7a were 5′-CCTGGATGTTCTCTTCACTG -3′ for sense and 5-GCCTGGATGCAGACTTTTCT-3′ for antisense sequences. 3 µl PCR mixture contained 0.5 µl of 10× PCR buffer, 0.7 µl of 25 mM MgCl2, 0.1 µl of 12.5 mM dNTPs, 0.01 µl UNG, 0.025 µl Amplitaq Gold DNA polymerase, 0.5 µl of dilute cDNA and water. PCR amplification consisted of 40 cycles (95°C for 30 s, 56°C to 60°C optimized for each primer set for 30 s and 72°C for 15 s) after the initial denaturation step (95°C for 10 min).

### Cytotoxicity Assay

After MCF-7 breast cancer cells (∼1.0×10^4^) were cultivated in 100 µl medium in the well of a 96-well plate for 12 h, the medium was discarded. 100 µl fresh medium alone or medium containing 20 µl of probe loaded CS/γ-PGA complex, probe loaded CS or probe loaded liposome was added to each well of the 96-well plate to cultivate for another 3 h. 20 µl of MTT (5 mg/ml) was then added to each well. After incubation for 4 h, the medium was removed and 150 µl of SDS (0.52 M) was added to each well to solubilize the formazan dye. After 15 min, the absorbance was measured using Hitachi/Roche System Cobas 6000 (Tokyo, Japan) at 490 nm, respectively. The relative cell viability (%) was calculated by (Atest/Acontrol)×100.

### In vivo Transfection

MDA-MB-231 and MCF-7 breast cancer cells were seeded on glass cover slides, which were put in DMEM containing ∼5×10^4^ cells per well in cell culture plates for 12 h, respectively. The media were then discarded, and l mL of fresh medium containing 20 µl of probe loaded CS/γ-PGA complex or control probe loaded CS/γ-PGA complex was added to each well and incubated for 3 h, respectively. Finally, the cover slides were taken out from the wells, rinsed thoroughly with sterile PBS buffer, and fixed on glass slides to detect the confocal images with a laser scanning confocal microscope (LSM-710 Zeiss, Germany).

### Apoptosis Experiments

The apoptosis experiments were carried out with AnnexinV/PI staining staining. After MCF-7 breast cancer cells (∼5×10^4^) were seeded on glass cover slides, the culture media were replaced with media containing 20 µl of probe loaded CS/γ-PGA complex for further 3 h. Afterwards, the cells were thoroughly washed and resuspended in fresh media to culture for another 24 h. The resulting cells were harvested, permeabilized with 96% ethanol and stained in 500 µl binding buffer supplemented for 10 min in dark. The cells were collected and analyzed by flow cytometry on FACSCalibar flow cytometer (Becton Dickinson, USA).

## Results and Discussion

### Characterization of Probe Loaded CS/γ-PGA Complex

**Figure 2:**
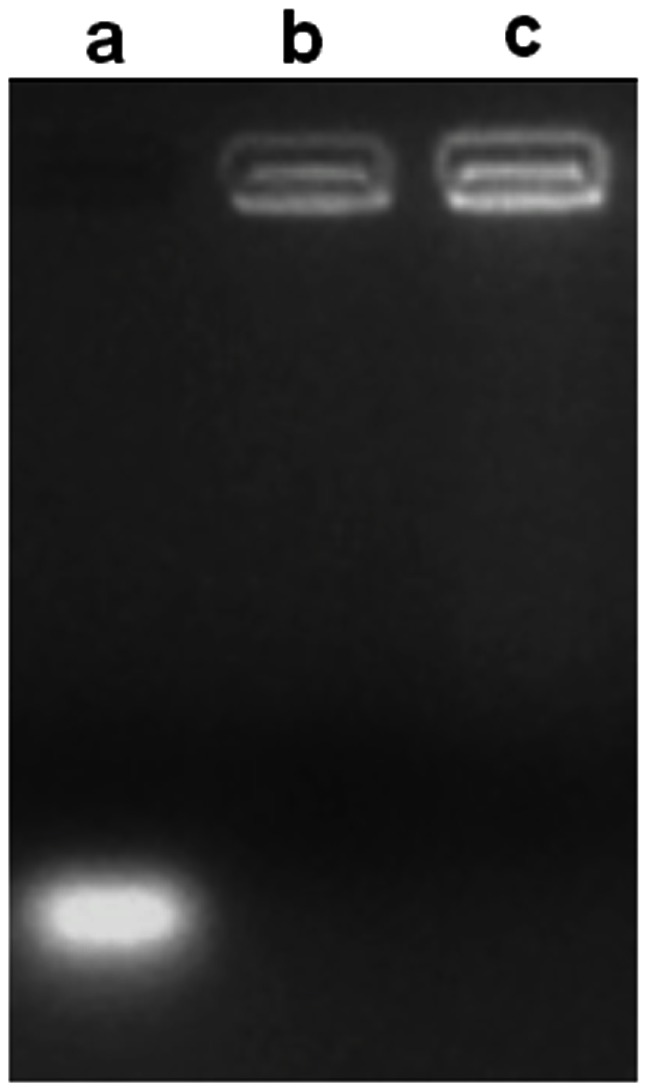
Gel retardation assay of NP probe complex. (a) QD-RNA-Au NP probe, (b) QD-RNA-Au NP probe loaded CS, and (c) QD-RNA-Au NP probe loaded CS/γ-PGA complex.

The binding capacity of QD-RNA-Au NP probe with CS/γ-PGA complex was evaluated using gel retardation assay. The probe showed a quick migration from the original position; while both the probe loaded CS and the probe loaded CS/γ-PGA complex did not show any migration (Figure 2). This result indicated the QD-RNA-Au NP probe could stably loaded on both CS and CS/γ-PGA complex. According to the previous report [29], after loading of DNA, the formed complex contained two domains, DNA/CS and CS/γ-PGA domains, the stable loading happened on CS domain (Scheme 1), and the presence of γ-PGA resulted in a significant increase in the cellular uptake due to its recognition to γ-glutamyl transpeptidase in the cell membrane. Therefore, the gel electrophoresis of probe loaded CS and CS/γ-PGA complex showed the same appearance. The stable loading resulted from the strong electrostatic interaction between the positively charged amino groups (−NH_3_^+^) of CS and the negatively charged phosphate groups (−PO4^−^) of RNA in pH 6.0 sodium acetate buffer. The formation of CS/γ-PGA complex was also due to the electrostatic interaction between the charged amino groups of CS and the –COO^–^ group of γ-PGA [28].

### In vitro Real-time PCR Detection of Pre-micRNA in Model Cells

**Figure 3:**
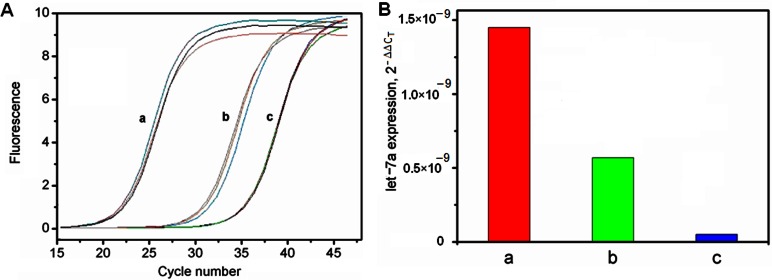
Real-time PCR detection of pre-micRNA let-7a expression. (a) normal human vascular endothelial cells, (b) MCF-7 and (c) MDA-MB-231 breast cancer cells.

The expression of pre-miRNA let-7a precursors in MCF-7 and MDA-MB-231 breast cancer cells was firstly profiled using in vitro real-time PCR detection. The cycle number at which the reaction crossed an arbitrarily placed threshold (*C*_T_) was determined for each gene, and the relative amount of pre-miRNA, normalized to β-actin reference and relative to a calibrator, was described using 2^−ΔΔC^_T_. [32] The *C*_T_ value generated from the MCF-7 cells was higher than that for normal human vascular endothelial cells, but lower than that for MDA-MB-231 cells (Figure 3A), which meant that the amount of pre-miRNA let-7a precursor in MCF-7 cells was higher than that in MDA-MB-231 cells (Figure 3B). They were 39.3% and 3.4% of the pre-miRNA let-7a precursor in normal human vascular endothelial cells, respectively, suggesting a potential association with the disease.

### In vitro Fluorescent Detection of Pre-microRNA Using the Designed Probe

**Figure 4:**
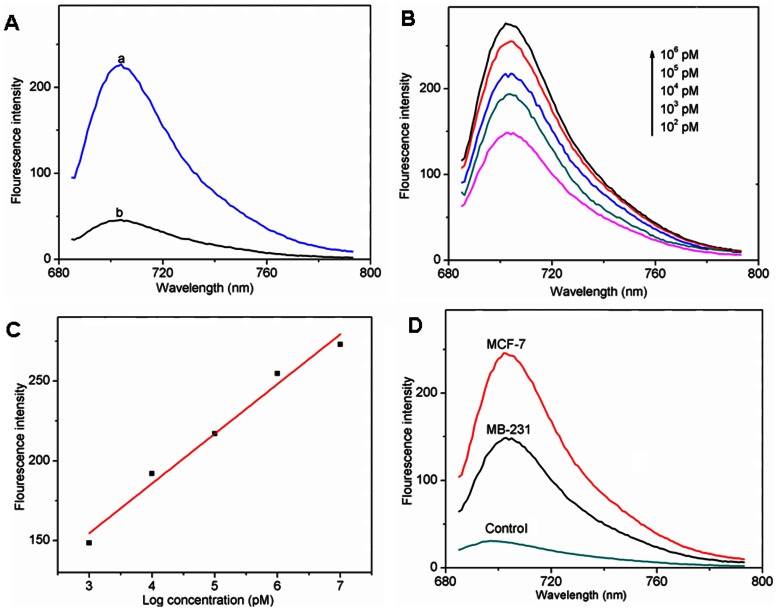
In vitro fluorescent detection of pre-microRNA. (A) Fluorescence spectra of (a) QD-RNA-Au NP and (b) QD-control RNA-Au NP probe loaded CS/γ-PGA complex in presence of target pre-miRNA. (B) Fluorescence spectra of QD-RNA-Au NP probe loaded CS/γ-PGA complex at different amounts of pre-miRNA and (C) linear plot of fluorescence intensity vs pre-miRNA amount. (D) Fluorescence spectra of the mixtures of QD-RNA-Au NP probe loaded CS/γ-PGA complex and 50 ng of total RNAs extracted from MCF-7 and MDA-MB-231 cells, 100 µl DEPC-treated water was used as control.

In order to demonstrate the capability of the QD-RNA-Au NP probe loaded CS/γ-PGA complex in monitoring pre-miRNA, in vitro fluorescent detection of pre-microRNA was performed after incubation of the probe loaded complex with target pre-miRNA and treatment with RNase III for 30 min. As shown in Figure 4A, the fluorescence spectrum showed a high intensity at the maximum emission wavelength of 705 nm (curve a), while the QD-control RNA-Au NP probe loaded complex showed much lower response (curve b), indicating the efficient binding of target miRNA with the matched probe and cleavage of the hybridization product, which led to the separation of QD from Au NP. The fluorescence intensity linearly increased with the increasing amount of target pre-miRNA from 0.2 µM to 2 mM (10^2^ to 10^6^ pmoL), suggesting a method for in vitro fluorescent detection of pre-miRNA (Figure 4B and 4C).

The probe loaded complex was further used to detect the pre-miRNA let-7a precursors in the samples extracted from both MCF-7 and MDA-MB-231 cells. As shown in Figure 4D, 50 ng of total RNA extracted from these cells showed clear fluorescence responses. Similar to the results from in vitro real-time PCR detection, the expression of pre-miRNA let-7a precursor in MCF-7 cells was higher than that in MDA-MB-231 cells. Thus, the QD-bound RNA probe could hybridize with target pre-miRNA in lysed cell sample, and the formed product could be cleaved by RNase III Dicer to separate the QDs and Au NPs, leading to the detectable fluorescence emission of QDs.

### In vivo Transfection and Detection of Pre-microRNA

**Figure 5:**
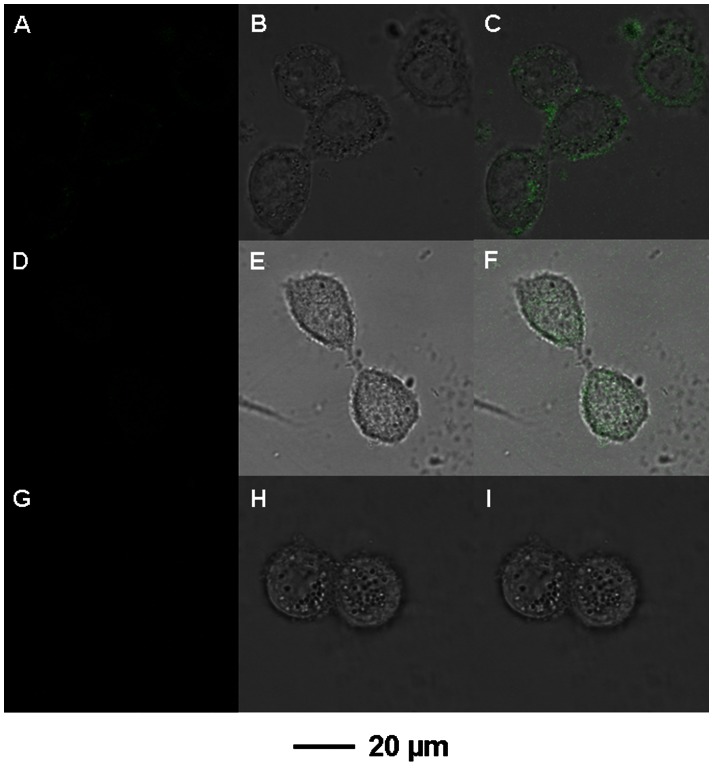
In vivo transfection and detection of pre-microRNA by confocal microscope. Confocal images of MCF-7 (A, B, C) and MDA-MB-231 (D, E, F) cells after transfected with QD-RNA-Au NP probe loaded CS/γ-PGA complex, and MCF-7 cells after transfected with QD-control RNA-Au NP probe loaded CS/γ-PGA complex (G, H, I) at 37°C for 3 h: fluorescence field (A, D, G), bright field (B, E, H) and overlapped images (C, F, I).

Confocal microscopic imaging was performed to assess the capability of the CS/γ-PGA complex as gene vector to transfer the probe, which possessed remarkable affinity and specificity to pre-miRNA target, into breast cancer cells of MCF-7 and MDA-MB-231 for the detection of pre-miRNA precursors. After 3-h transfection, QD-RNA-Au NP probe loaded CS/γ-PGA complex was delivered into the cells, and released the probe into the cytoplasm by electrostatic repulsion at intracellular pH. The released probe then hybridized with pre-miRNA precursor as target, and the formed product was cleaved by RNase III Dicer to separate QD from Au NP. Thus the confocal microscopic image revealed the fluorescence of the QD (Figure 5).

The fluorescent intensity depended on the amount of pre-miRNA in cytoplasm when the same concentration of probe loaded complexe was used in the transfection process, producing a method for specific detection of pre-miRNA due to the specific recognition of probe to pre-miRNA.

**Figure 6:**
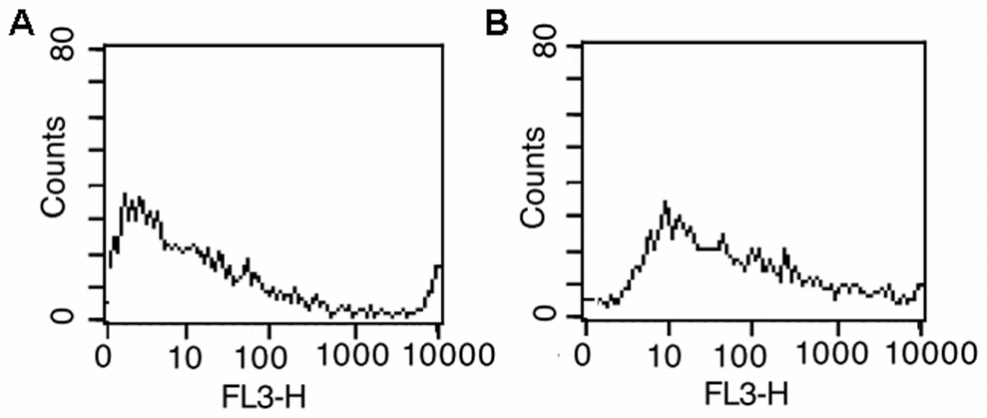
Flow cytometric analysis. (A) MCF-7 and (B)MDA-MB-231 cells after transfected with probe loaded complex.

The MCF-7 cells transfected with the probe loaded complex (Figure 5C) exhibited much stronger intensity than those transfected with MDA-MB-231 cells (Figure 5F). The control probe loaded complex was no fluorescence in MCF-7 cells (Figure 5I). From the confocal microscopic image, the mean values of fluorescence obtained from transfected MCF-7 and MDA-MB-231 cells were 147.5 and 86.3, respectively, producing a ratio of the fluorescence intensities of 1.7∶1. This result was comparable to the ratio of fluorescence intensities of 1.4∶1 obtained with flow cytometric analysis (Figure 6).

### Cytotoxicity

**Figure 7:**
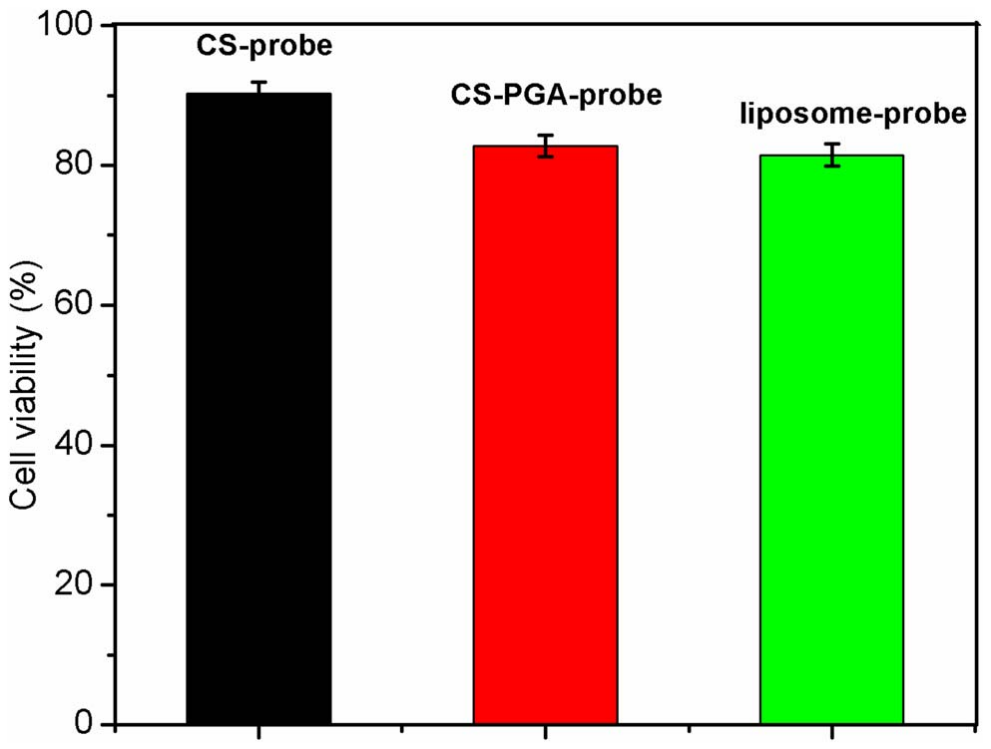
Cytotoxicity induced by QD-RNA-Au NP probe loaded CS, CS/γ-PGA complex and liposome in MCF-7 cells.

The cytotoxicity depends on the biocompatibility of the gene vector. MTT assay was carried out to evaluate the cytotoxicity of the QD-RNA-Au NP probe loaded CS/γ-PGA complex. As comparison, the cytotoxicity of CS and liposome was also investigated with the same procedure, respectively. As shown in Figure 7, after MCF-7 breast cancer cells were cultivated in medium containing the probe loaded CS, CS/γ-PGA complex and liposome for 3 h, the CS/γ-PGA transfected cells exhibited 83.7% viability, while the CS and liposome-transfected cells showed 92.3% and 81.5% viability, respectively, indicating lower cytotoxicity of CS/γ-PGA than liposome in MCF-7 cells. Thus CS/γ-PGA complex could be a potential gene vector for future application.

**Figure 8:**
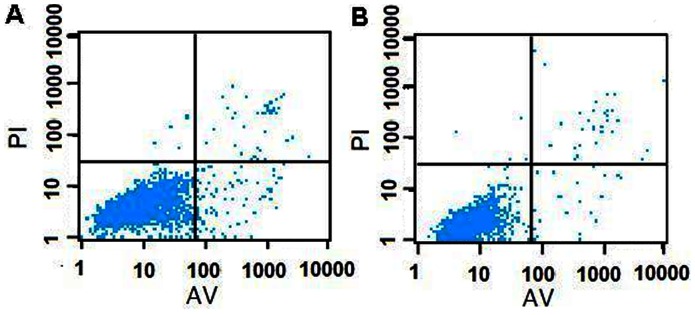
Flow cytometric apoptotic analysis. (A) MCF-7 cells and (B) MCF-7 cells transfected with probe loaded complex at 37°C for 3 h.

The apoptosis of MCF-7 breast cancer cells induced by CS/γ-PGA complex was monitored with flow cytometric analysis by AnnexinV/PI staining. The control group that was incubated in absence of the probe loaded complex presented an apoptotic ratio of 2.61% (Figure 8A), while the cells transfected with the probe loaded complex displayed slightly higher apoptotic ratio of 5.37% (Figure 8B), which further suggested CS/γ-PGA/probe could efficiently remain viability.

### Conclusion

A novel strategy was presented for both in vitro detection of miRNA using a newly designed QD-RNA-Au NP probe and in vivo detection of intracellular pre-miRNA using CS/γ-PGA complex as a gene vector. The delivery system showed high transfection efficiency and could transfer FRET-based probe into the cells to recognize the target pre-miRNA, which produced a method for in situ detection of intracellular pre-miRNA. The strategy was demonstrated using the pre-miRNA recognized and cleaved system in the cytoplasm. The probe loaded CS/γ-PGA complex provided a robust, sensitive, and selective sensor for detection of target pre-miRNA. Using breast cancer cells (MCF-7, MDA-MB231) as models, the CS/γ-PGA showed negligible cytotoxicity and improved the sensitivity of pre-miRNA detection. This new strategy for detection of pre-miRNA is expected to hold promising application potential for medical research.
